# The Clinical Impact of Methylated Homeobox A9 ctDNA in Patients with Non-Resectable Biliary Tract Cancer Treated with Erlotinib and Bevacizumab

**DOI:** 10.3390/cancers14194598

**Published:** 2022-09-22

**Authors:** Line Bechsgaard Andersen, Marit Sofie Kjær Mahler, Rikke Fredslund Andersen, Lars Henrik Jensen, Louise Raunkilde

**Affiliations:** 1 Faculty of Health, University of Southern Denmark, 5000 Odense, Denmark; 2 Department of Oncology, Vejle Hospital, University Hospital of Southern Denmark, 7100 Vejle, Denmark; 3 Department of Biochemistry and Immunology, Vejle Hospital, University Hospital of Southern Denmark, 7100 Vejle, Denmark; 4 Institute of Regional Health Research, University of Southern Denmark, 5000 Odense, Denmark

**Keywords:** biliary tract cancer (BTC), cholangiocarcinoma, circulating tumor DNA (ctDNA), DNA methylation, HOXA9, droplet digital polymerase chain reaction (ddPCR), erlotinib, bevacizumab, adverse events

## Abstract

**Simple Summary:**

Non-resectable biliary tract cancer is incurable. The balance between last-line treatment, with limited improvement in survival, and potential adverse events calls for prognostic biomarkers aiding the decision making process. The aim of this retrospective study was to investigate the clinical impact of the circulating tumor DNA (ctDNA), methylated homeobox A9, in plasma from 39 patients receiving erlotinib and bevacizumab for non-resectable biliary tract cancer. Treatment effect and adverse events were also investigated. The study found an increase in ctDNA after one treatment cycle implying that the biomarker is negatively associated with survival in patients with late stage BTC.

**Abstract:**

Methylated homeobox A9 (meth-HOXA9) is tumor specific and has been suggested as a prognostic biomarker in several types of cancer. ctDNA measured as meth-HOXA9 may be a valuable biomarker in the decision-making process about last-line treatment of biliary tract cancer (BTC). The aim of the study was to investigate the clinical impact of meth-HOXA9 in plasma from patients receiving erlotinib and bevacizumab for late-stage BTC and to investigate the treatment effect and adverse events. Droplet digital PCR was applied to detect meth-HOXA9 in 39 patients. Response rates were registered according to RECIST (1.1) and adverse events according to Common Terminology Criteria for Adverse Events Version 4.0 (CTCAE (4.0)). Endpoints were progression-free survival (PFS), overall survival (OS), response rate, and toxicity. A significant difference in PFS and OS between patients with increasing and non-increasing meth-HOXA9 was detected after one treatment cycle, hazard ratio (HR) 12.4 (*p* < 0.0001) and HR 2.75 (*p* = 0.04), respectively. The most common adverse events of erlotinib were fatigue, pain, and rash, and those of bevacizumab were bleeding and wounds. This study found meth-HOXA9 to be negatively associated with survival in patients with late-stage BTC. Hence, meth-HOXA9 may guide early discontinuation of ineffective treatment.

## 1. Introduction

Biliary-tract cancer (BTC) is a relatively rare disease comprising less than 1% of all cancers [1]. The incidence and mortality are increasing and the disease is one of the most fast growing cancers in the world [2]. BTC is often detected at a late stage and is often non-resectable at the time of diagnosis. Consequently, survival of non-resectable BTC is poor with a median overall survival (OS) of about 10–12 months, and the aim of treatment is most often palliative [3,4]. The first-line treatment of non-resectable BTC is combination chemotherapy with gemcitabine and cisplatin [2,5], and recently a phase 3 study of the doublet chemotherapy in combination with the anti-PD-L1 agent durvalumab, showed improved overall survival [6]. There is no standard last-line treatment, but patients in good performance can be offered experimental therapy and, alternatively, best supportive care. In Denmark, experimental therapy has been offered with the tyrosine kinase inhibitor erlotinib and bevacizumab, inhibiting the epidermal growth factor receptor (EGFR) and the vascular endothelial growth factor (VEGF), respectively. [7,8]. However, studies indicate limited benefit of erlotinib and bevacizumab, prolonging life with months rather than years [9,10].

Erlotinib and bevacizumab treatment may be associated with significant adverse events. Two meta-analyses of BTC patients receiving erlotinib found the most common adverse events to be dermatological toxicity and diarrhea [11,12]. In a randomized phase II trial of patients with renal cell carcinoma receiving bevacizumab (vs. placebo) hypertension, proteinuria, and epistaxis were the predominant adverse events [13]. Another study on bevacizumab in colorectal cancer (CRC) found 2% and 13% of the patients to develop bowel perforation and thrombosis, respectively [14]. 

An average small effect on survival of erlotinib and bevacizumab, together with potentially significant adverse events calls for biomarkers predictive of effect and prognosis. A biomarker could be instrumental in deciding whether treatment should be started and an early indicator of treatment effect, thereby enabling termination of ineffective toxic treatment at an early time. 

Aberrantly methylated circulating tumor DNA (ctDNA) has been suggested as a prognostic and predictive marker, since aberrant methylation may represent an early event in cancer tumorogenesis [15,16,17,18,19,20]. Some studies have found prognostic value of circulating cell-free DNA (cfDNA) or ctDNA in patients with metastatic CRC [21,22]. The specific ctDNA, methylated Homeobox A9 (meth-HOXA9), has been found in several studies to be associated with prognosis and treatment effect. The HOXA9 gene encodes a DNA-binding transcription factor regulating gene expression, but its specific function is unknown [23]. A study including patients with late-stage, non-small cell, lung carcinoma (NSCLC) found meth-HOXA9 to be a negative prognostic factor at baseline with enhanced impact after the first treatment cycle [24]. Another study on NSCLC reported similar results [25]. Rusan et al. [26] measured meth-HOXA9 during treatment with PARP inhibitors in ovarian cancer. They found, during multiple cycles, worse clinical outcomes in patients with detectable meth-HOXA9 compared to those with undetectable meth-HOXA9, suggesting meth-HOXA9 as a valuable predictive biomarker.

Well-known prognostic factors of BTC are tumor-node-metastasis (TNM) classification along with the anatomical location of the cancer [1,7,27]. The non-invasive approach detecting meth-HOXA9 in blood may contribute with prognostic value and to our knowledge; the issue has not previously (even preclinical) been investigated in relation to late stage BTC. 

The aim was to investigate the clinical impact of meth-HOXA9 in plasma from patients receiving erlotinib and bevacizumab for non-resectable BTC and, furthermore, to investigate the effect and adverse events of the treatment.

## 2. Materials and Methods

### 2.1. Patient Eligibility 

This study was a retrospective analysis of a prospectively collected cohort recruited at the Department of Oncology, Vejle Hospital, Denmark, in the period 2012 to 2017. Patients enrolled had previously undergone treatment with combination chemotherapy, and experimental treatment with erlotinib and bevacizumab was offered at progression. 

The inclusion criteria were metastatic or locally advanced incurable BTC, treatment with erlotinib and bevacizumab indicated, measurable disease on CT scan within 4 weeks from inclusion, age ≥ 18 years, and orally and written informed consent. The exclusion criteria were pregnant and breast-feeding women, unwillingness by fertile patients to use contraception, and contraindications to erlotinib or bevacizumab.

### 2.2. Treatment 

Patients received oral erlotinib 150 mg daily along with intravenous bevacizumab 10 mg/kg every two weeks. Treatment was given until progression, unacceptable toxicity despite dose modifications, or until patients requested discontinuation. 

Unacceptable toxicity was defined as grade 3 adverse events or above in accordance with the Common Terminology Criteria for Adverse Events version 4.0 (CTCAE 4.0) [28]. In these cases, and in the case of low-grade adverse events untreatable with supportive care, gradual dose reduction of erlotinib by 50 mg was performed or the treatment was discontinued.

### 2.3. Endpoints 

The primary objective was to investigate the association with survival of meth-HOXA9 in late-stage BTC patients treated with erlotinib and bevacizumab and to describe the effect and adverse events of the treatment. The endpoints of the study were progression-free survival (PFS), overall survival (OS), response rate, and toxicity. 

### 2.4. Efficacy 

Response was evaluated radiologically every 8–12 weeks according to the Response Evaluation Criteria in Solid Tumours version 1.1 (RECIST 1.1) [29]. Patients were clinically assessed every 2–4 weeks and adverse events related to erlotinib and bevacizumab were graded by a doctor according to CTCAE (4.0), noted on a paper-based case report form (CRF) and saved in the patient record. All paper based CRFs were double checked, comparing the adverse events with those written in the electronic medical records. In five cases, the paper based CRF on adverse events was missing, and information on those was based on the electronic medical records. 

### 2.5. Analysis of Methylated HOXA9 

Blood samples were collected at baseline and at every treatment cycle and stored in a biobank until analysis in 2020. For every sample, 4 mL plasma was isolated by centrifugation.

Purification proceeded using QIAsymphony DSP Circulating DNA kit (Qiagen, Hilden, Germany). DNA was eluted in a standard volume of 60 µL. To secure quality control, samples were analyzed by quantitative PCR (Quantstudio 12K Flex, Thermo Fisher Scientific, Waltham, MA, USA). DNA extraction was assessed by CPP1 (intern exogenous control) and β-2 microglobulin (a marker for the amount of DNA purified in every sample) [30]. The DNA was bisulfite-converted with EZ DNA methylation lightning kit (Zymo research, Irvine, CA, USA). As fully specified by Wen et al. [24], three positive and negative controls for the bisulfite conversion were used. A fourth assay control (EpiTect Control DNA, Qiagen, Hilden, Germany), which was purchased as bisulfite-converted DNA, was included in the final droplet digital PCR (ddPCR) analysis.

The converted DNA was analyzed by ddPCR with HOXA9 methylated specific assay and albumin assay distributed in two 20 µL reactions. Albumin was used as a reference gene [26]. The four controls were analyzed by ddPCR parallel with the patient sample. 

The result from the analysis was read by QX200 Droplet Digital Reader (Bio-Rad, Hercules, CA, USA). Samples were classified as positive if >4 meth-HOXA9 positive droplets were detected in the analysis. Data were reported using QuantaSoft^TM^ (Bio-Rad, Hercules, CA, USA) as the number of [meth-HOXA9 copies/reaction]/[Albumin copies/reaction] × 100 with a 95% confidence interval (CI) derived from a Poisson distribution [31]. 

Meth-HOXA9 was considered undetectable, if the lower 95% CI included zero. The dynamics of meth-HOXA9 during treatment was considered increasing if the measurement was above the 95% CI of baseline value and decreasing if below the 95% CI of baseline value. Meth-HOXA9 was considered changed if its status went from detectable to undetectable or vice versa. 

### 2.6. Statistical Analysis 

The study was an explorative and descriptive trial. Patients undergoing treatment for at least two and four weeks were evaluable for toxicity and response, respectively. All enrolled patients were evaluable for PFS and OS according to the intention-to-treat principle. PFS and OS were calculated from the day of enrollment to the date of progression and death from any cause, respectively, or censored at the last hospital contact. At the end of the first treatment cycle PFS and OS of the patients with increasing meth-HOXA9 were compared with the patients with non-increasing meth-HOXA9 and were included in the analysis on the date the second treatment cycle was started.

Patient characteristics and levels of meth-HOXA9 were compared with Fisher’s Exact test or chi-squared test for categorical variables and Wilcoxon rank-sum test for numeric variables.

Kaplan–Meier curves illustrated the association of meth-HOXA9 with PFS and OS. Comparison was undertaken using the Log-rank test. Hazard ratios (HR) were calculated using COX regression analysis with 95% CI. A *p*-value of ≤0.05 was considered statistically significant.

Meth-HOXA9 was measured as a binary parameter (detectable and undetectable) at baseline and continuously (changes in copies/mL) at every treatment cycle. Statistical calculations were performed using Stata/BE 17.0 (StataCorp LLC, College Station, TX, USA).

### 2.7. Ethical Considerations 

The study was conducted in accordance with the Declaration of Helsinki, and the protocol was approved by The Regional Committee on Health Research Ethics for Southern Denmark (S-20120101). Informed consent was obtained from all subjects involved in the study. 

## 3. Results

### 3.1. Patient Characteristics

From July 2012 to October 2017, 39 patients were enrolled. Baseline characteristics and status according to dynamics in meth-HOXA9 are shown in Table 1. The median age was 62 years with an equal distribution of men and women. At the time of enrollment, the majority of the patients were in ECOG performance status (PS) 1 (67%). All patients had received at least one previous treatment line and one had undergone curatively intended surgery. A total of 28 patients (72%) had metastatic disease and meth-HOXA9 was detectable in 37 patients (95%). Subtypes of BTC and the median meth-HOXA9 levels at baseline are shown in Table 2. The majority of patients had an intrahepatic BTC (*n* = 23). These patients also had the highest median level of meth-HOXA9 in plasma at baseline (206 copies/mL). 

### 3.2. Meth-HOXA9—Dynamics and Survival 

Thirty-nine patients were evaluable for meth-HOXA9 analysis at baseline. Furthermore, 33 and 18 patients were evaluable for analysis at the beginning of the second and the third cycle of treatment, respectively. There was no significant difference between the methylation groups (increasing and non-increasing) regarding patient characteristics except from PS.

**Figure 1:**
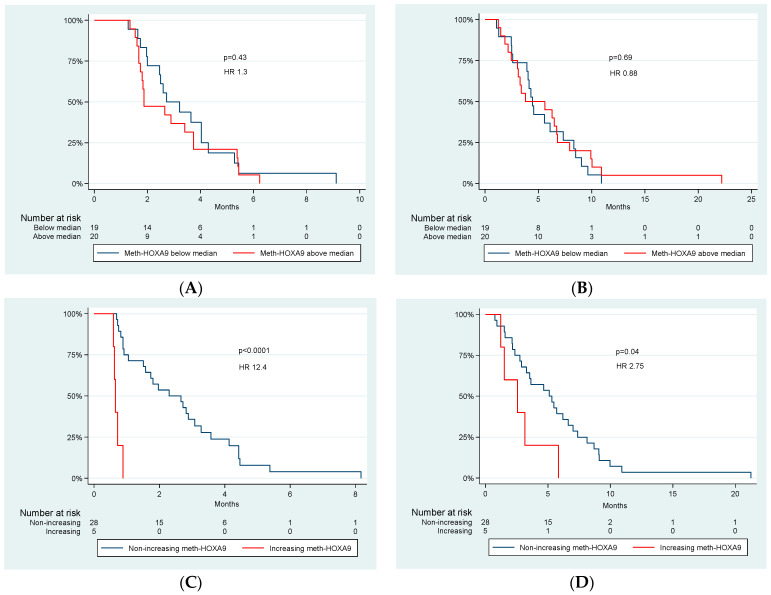
Kaplan-Meier plots for meth-HOXA9 below median vs. meth-HOXA9 above median at baseline ((**A**) PFS at baseline, (**B**) OS at baseline) and non-increasing meth-HOXA9 vs. increasing meth-HOXA9 after one treatment cycle ((**C**) PFS after one treatment cycle, (**D**) OS after one treatment cycle). *p*-values and hazard ratios (HR) are shown.

The median PFS and OS of all patients was 2.7 and 4.5 months, respectively. There was no significant difference in PFS and OS between patients with detectable and undetectable meth-HOXA9 at baseline (HR 0.38, *p* = 0.18) and (HR 1.02, *p* = 0.98), respectively. Comparing patients with high and low levels of meth-HOXA9 at baseline (above and below the median) no significant difference was found (Figure 1A,B).

After the first treatment cycle meth-HOXA9 was detectable in 82% of patients (28 vs. 5 patients), i.e., the 95% CI did not include zero, but no significant difference in PFS and OS between detectable and undetectable meth-HOXA9 could be shown at that time point. 

Patients with an increase (*n* = 5) in meth-HOXA9 after the first treatment cycle had a median PFS of 0.7 months compared to 2.3 months in patients with stable or decreasing meth-HOXA9 (non-increasing, *n* = 28) (Figure 1C). The difference was significant; HR = 12.4 (95% CI: 3.63–42.25, *p* < 0.0001). This strong association was supported by a significant increase in OS after one treatment cycle in patients with non-increasing meth-HOXA9; HR = 2.75 (95% CI: 1.00–7.55, *p* = 0.04), (Figure 1D). After one treatment cycle the median OS of patients with increasing and non-increasing meth-HOXA9 was 2.6 and 5.1 months, respectively. The negative association also applied to PFS after the second treatment cycle (the beginning of the third treatment cycle); HR = 5.1 (95% CI: 1.46–17.71, *p* = 0.0047). Again, the PFS result was supported by a significant difference in OS; HR = 4.11 (95% CI: 1.28–13.21, *p* = 0.0105).

### 3.3. Response

Treatment response was evaluable in 32 patients (partial response = 1, stable disease = 17, progression = 14). No patients had complete response.

### 3.4. Adverse Events and Meth-HOXA9

Of the 39 enrolled patients 37 were evaluable for toxicity. The most common adverse events were fatigue (86%), pain (70%), rash (62%), loss of appetite (62%), infection (51%), and nausea (43%) (Table 3). 

Grade 3/4 and a maximum of grade ≤ 2 adverse events were registered in 14 (38%) and 23 patients (62%), respectively. Of the 14 patients, only four stopped treatment due to adverse events. Grade 3 adverse events included infection (19%), pain (11%), and fatigue (5%). Three patients experienced grade 4 adverse events, which caused two of them to discontinue. Adverse events associated to treatment with bevacizumab were bleeding (21%) and wound (5%). None of the four patients who stopped treatment due to adverse events had an increase in meth-HOXA9 after the first treatment cycle. There was no correlation between increasing meth-HOXA9 and grade 3–4 adverse events (*p* = 0.67). Additionally, the respective minor adverse events were not associated with changes in HOXA9.

## 4. Discussion

To the best of our knowledge this is the first study to explore the clinical value of meth-HOXA9 in late stage BTC in patients treated with erlotinib and bevacizumab. The baseline level of meth-HOXA9 was not informative, but a strong impact of meth-HOXA9 was found already after one treatment cycle. The outcome of patients with increasing meth-HOXA9 was worse than that of patients with a non-increasing level; HR = 12.4 and 2.75 for PFS and OS, respectively. This implies that an early increase in meth-HOXA9 yields a worse prognosis, and meth-HOXA9 may be a useful tool in regard to early stop of ineffective treatment of BTC.

In a review, several studies found ctDNA to be a promising clinical biomarker in relation to BTC [32]. Other studies have found a prognostic value of ctDNA or cfDNA in plasma. Jensen et al. [33] found significantly improved survival in BTC patients with low total plasma DNA and a trend toward improved PFS and OS in patients with non-detectable KRAS mutation in plasma. Studying 123 patients with metastatic CRC, Thomsen et al. [22] found a low level of methylated ctDNA after one cycle of treatment implied increased PFS and OS. Steffensen et al. [34] reported a prognostic value of cfDNA, finding increased PFS and OS in late stage ovarian cancer patients with a low baseline level of cfDNA (below the median).

Narrowing down to meth-HOXA9, a study analyzed the clinical impact of meth-HOXA9, Homeobox D9, and Opioid Binding Protein/Cell Adhesion Molecule Like (OPCML) in BTC patients [35]. The latter two were found to be promising biomarkers, but only for differential diagnostics of BTC and other biliary diseases. Other studies have found a prognostic value of meth-HOXA9. Rusan et al. [26] reported a negative relation between detected meth-HOXA9 and PFS and OS after three treatment cycles in patients with BRCA-mutated ovarian cancer. Analyzing patients with recurrent ovarian cancer Thomsen et al. [36] reported a prognostic value after one treatment cycle, when comparing patients with increasing and stable meth-HOXA9 levels. However, the cohort sizes of the two above phase II studies [26,36] were rather small (*n* = 32 and *n* = 23, respectively) as in the present study. Jakobsen et al. [37] found a prognostic impact of meth-HOXA9 in ovarian cancer and late-stage NSCLC patients. This was supported by Wen et al. [24] reporting a prognostic impact at baseline and after one treatment cycle analysing 231 late-stage NSCLC patients.

Most studies analysing meth-HOXA9 applied the established ddPCR method in detecting DNA in plasma, but the retrospective nature and the relatively small cohorts of some studies, including the present one, affect the validity of the findings. The majority of the mentioned studies reported a prognostic impact of detectable ctDNA or meth-HOXA9 in binary analyses whereas our study found a significant impact only when comparing patients with and without an increase in meth-HOXA9. 

Only one patient had partial response in the present study and the median PFS and OS of all patients of 2.7 and 4.5 months, respectively, indicates minimal effect of the treatment. These findings correspond to those of other studies investigating the clinical effect of erlotinib and bevacizumab [9,10,38] and emphasize the importance of considering potential adverse events in decisions on last-line treatment. 

The most common adverse events were as expected and comparable to the findings of other studies [10,11,12,39,40,41]. There was no association between adverse events and dynamics of meth-HOXA9 after the first treatment cycle. 

Most of the patients in the present study had undergone one or more treatment lines previously and were relatively heavily pre-treated at the time of enrollment. Additionally, they had late-stage disease, and although the majority had a good initial PS, the disease rapidly developed in some patients. This may have had some effect on the treatment outcome and adverse events. Patients with increasing and non-increasing meth-HOXA9, respectively, did not differ significantly in any baseline parameter except for PS, but the subgroup of increasing meth-HOXA9 was small and calculations should be performed with caution.

Our study is limited by the small population size and its retrospective nature. Storage of blood samples for a long time may cause the cfDNA to degrade and result in a lower detection rate of meth-HOXA9 in plasma [42]. However, the median of total DNA copies/mL in this study was comparable to that of other similar, more recent cohorts, implying that degradation of DNA in the samples most likely did not occur. This was supported by 37 of 39 patients having detectable meth-HOXA9 at baseline. Since the cohort consists of Caucasians, results may not be fully applicable to other ethnic groups. 

The application of the well-known and reproducible ddPCR method for the analysis of meth-HOXA9 in plasma is a strength of our study [43,44], as well as the standardized collection of adverse events data according to CTCAE and radiological evaluation according to RECIST. Additionally, the use of liquid tissue biopsy is ethically and practically an advantage due to the minimally invasive procedure.

This study found a negative association between survival and increasing meth-HOXA9, valuable information on adverse events of erlotinib and bevacizumab, and best response rates of last-line treatment showing a limited number of patients with improvement in the disease. The majority of the patients experienced acceptable adverse events (grade 1 and 2), but, in the end, acceptance is subjective and varies among patients. 

At present, the initiation of last-line treatment of late-stage BTC is balanced between potential effect and risk of adverse events. In addition to imaging, future validated studies of meth-HOXA9 as a biomarker may add valuable information in the shared decision-making process, elucidating the need for start of treatment and for early discontinuation of ineffective treatment, respectively. The clinical application of meth-HOXA9, however, requires further prospective randomized studies with large cohorts. 

## 5. Conclusions

An early increase in meth-HOXA9 after one treatment cycle was negatively associated with survival in patients with late stage BTC, and the marker may be a useful tool in regard to early stop of ineffective treatment. Moreover, meth-HOXA9 may contribute with valuable information in the shared decision-making process, considering the balance between treatment effect and potential adverse events. We have shown clinical relevance of measuring ctDNA in BTC using meth-HOXA9, and suggest application of the marker in future trials evaluating treatment effect in BTC. 

## Figures and Tables

**Table 1:** Patient Characteristics and Dynamics of Meth-HOXA9 (*n* = 39).

Patient Characteristics	All Patients *n* = 39	Increasing Meth-HOXA9 after 1st Cycle (*n* = 5)	Non-Increasing Meth-HOXA9 after 1st Cycle (*n* = 28)	*p*-Value
Age at first treatment, years Median Range	62 25–80	71 55–73	64 25–80	0.24
Sex Male Female	19 (49%) 20 (51%)	2 (40%) 3 (60%)	14 (50%) 14 (50%)	0.53
Performance status 0 1 2	10 (25%) 26 (67%) 3 (8%)	4 (80%) 1 (20%) 0 (0%)	4 (14%) 22 (79% 2 (7%)	0.01
Metastatic or localized disease Metastatic Localized	28 (72%) 11 (28%)	4 (80%) 1 (0%)	20 (71%) 8 (29%	0.58
Tumorlocalization Intrahepatic Extrahepatic Gall bladder Unknown	23 (59%) 6 (15%) 1 (3%) 9 (23%)	3 (60%) 2 (40%) 0 (0%) 0 (0%)	17 (61%) 3 (10%) 1 (4%) 7 (25%)	0.28
Curative surgery previous Yes No	1 (3%) 38 (97%)	0 (0%) 5 (100%)	1 (4%) 27 (96%)	0.85
Type of previous therapy Gemcitabine Oxaliplatin Capecitabine Cisplatin Panitumumab Bevacizumab	39 (100%) 30 (77%) 28 (72%) 8 (21%) 5 (13%) 10 (26%)	5 (100%) 3 (60%) 3 (60%) 2 (40%) 0 (0%) 2 (40%)	28 (100%) 21 (75%) 21 (75%) 6 (21%) 4 (14%) 6 (21%)	0.46
HOXA9 Detectable Undetectable	37 (95%) 2 (5%)	5 (100%) 0 (0%)	26 (93%) 2 (7%)	0.71

**Table 2:** Tumorlocalization and Meth-HOXA9.

Tumorlocalization	Median Meth-HOXA9 Copies/mL Plasma at Baseline
Intrahepatic (*n* = 23)	206
Extrahepatic (*n* = 6)	12
Gall bladder (*n* = 1)	32
Unknown (*n* = 9)	19

**Table 3:** Adverse events of erlotinib and bevacizumab in Patients with Biliary Cancer (*n* = 37).

		*CTCAE Grade,* *N (%)*				
*Adverse* *Event*	0	1	2	3	4	Total 1–4
***Erlotinib***						
*Nausea*	21 (57%)	13 (35%)	2 (5%)	1 (3%)	0 (0%)	16 (43%)
*Vomiting *	29 (78%)	5 (14%)	1 (3%)	2 (5%)	0 (0%)	8 (22%)
*Loss of appetite *	14 (38%)	19 (51%)	4 (11%)	0 (0%)	0 (0%)	23 (62%)
*Diarrhea *	21 (57%)	9 (24%)	6 (16%)	1 (3%)	0 (0%)	16 (43%)
*Cough *	30 (81%)	6 (16%)	1 (3%)	0 (0%)	0 (0%)	7 (19%)
*Dyspnea*	32 (86%)	4 (11%)	1 (3%)	0 (0%)	0 (0%)	5 (14%)
*Fatigue *	5 (14%)	21 (57%)	9 (24%)	2 (5%)	0 (0%)	32 (86%)
*Stomatitis *	30 (81%)	7 (19%)	0 (0%)	0 (0%)	0 (0%)	7 (19%)
*Pain *	11 (30%)	12 (32%)	10 (27%)	4 (11%)	0 (0%)	26 (70%)
*Rash *	14 (38%)	12 (32%)	11 (30%)	0 (0%)	0 (0%)	23 (62%)
*Conjunctivitis *	35 (95%)	2 (5%)	0 (0%)	0 (0%)	0 (0%)	2 (5%)
*Infection *	18 (48%)	6 (16%)	4 (11%)	7 (19%)	2 (5%)	19 (51%)
*Interstitiel lung disease *	36 (97%)	0 (0%)	0 (0%)	0 (0%)	1 (3%)	1 (3%)
*** Bevacizumab ***						
*Bleeding *	29 (78%)	6 (16%)	0 (0%)	2 (5%)	0 (0%)	8 (21%)
*GI perforation *	37 (100%)	0 (0%)	0 (0%)	0 (0%)	0 (0%)	0 (0%)
*Arterial Thrombosis *	37 (100%)	0 (0%)	0 (0%)	0 (0%)	0 (0%)	0 (0%)
*Wound *	35 (95%)	2 (5%)	0 (0%)	0 (0%)	0 (0%)	2 (5%)

CTCAE, Common Terminology Criteria for Adverse Events.

## Data Availability

Patient data in this study are not publicity available due to Danish Data Legislation preventing disclosure of sensitive data.
